# Basilar Tip Aneurysm in Takayasu Arteritis

**DOI:** 10.1259/bjrcr.20180114

**Published:** 2019-11-15

**Authors:** Saima Ahmad

**Affiliations:** 1 Consultant Radiologist, Lahore General Hospital, Lahore, Pakistan

## Abstract

Takayasu arteritis is a chronic, inflammatory, progressive and idiopathic disease that mainly effects the aorta, its branches and pulmonary artery. It causes narrowing and occlusion of major vessels which manifest as a cerebrovascular insufficiency. Intracranial aneurysms are rarely observed in these patients. We report a case of basilar tip aneurysm with Takayasu arteritis. To our knowledge only 26 cases of Takayasu disease with basilar aneurysm have been reported in the literature. Management of Takayasu disease includes immunosuppression therapy; but coil embolization of co-incidental aneurysm can be hampered by limited and tortuous access routes. Hence, management of these patients requires great care and accurate selection of suitable guiding catheter, microcatheters and microwires. Microsurgery is the treatment of choice in case of intracranial aneurysm due to limited and tortuous access routes, however, coil embolization can be done with great care and accurate selection of suitable guiding catheter, microcatheters and microwires.

## Introduction

Subarachnoid hemorrhage secondary to ruptured intracranial aneurysms is a life threatening condition with an incidence of 6 to 7 per 100,000 person–years in many populations.^1^ The overall prevalence was estimated as 3.2% [95% CI (1.9–5.2)] in a population without comorbidity, with a mean age of 50 years, and consisting of 50% males.

Takayasu arteritis (TA) is a chronic, large vessel vasculitis of unknown etiology that typically effects aorta and its branches.^2^ It was first reported in 1908 by Takayasu, a Japanese ophthalmologist.^3^ Intracranial involvement in the form of stenosis and occlusion of proximal cerebral arteries have been reported in 24% of the cases of aorto-arteritis.^2^ Isolated case reports of intracranial aneurysms associated with TA are mostly reported in Japan.

Hemodynamic stress caused by obstruction of cervical vessels develop cerebral aneurysms in these patients and they involve the posterior circulation more often than aneurysms in the general population.^4^ The purpose of this study is to report the first diagnosed case of Takayasu arteritis with intracranial aneurysm in Pakistan.

## Case report

The 32-year-old female was referred to the center for vascular consultation. The patient was a house wife and a mother of two children. She reported having an episode of sudden onset severe headache associated with generalized tonic and clonic fits and loss of consciousness. She regained consciousness after a period of 24 h without any neurological deficit with a complaint of neck rigidity and headache. The patient had a past history of similar episodes dating back 1 year. Upon interview she recalled a long history of trivial headaches, malaise and low grade fever for the past 6 years, whose severity had never warranted any further investigation. She also had a history of intermittent claudication in her bilateral upper limbs. Physical examination revealed no neurological deficit, however, radial and brachial arteries were impalpable bilaterally. Right to left blood pressure discrepancies were detected, measuring at 140/80 from right arm and 160/80 from the left arm.

**Figure 1.**
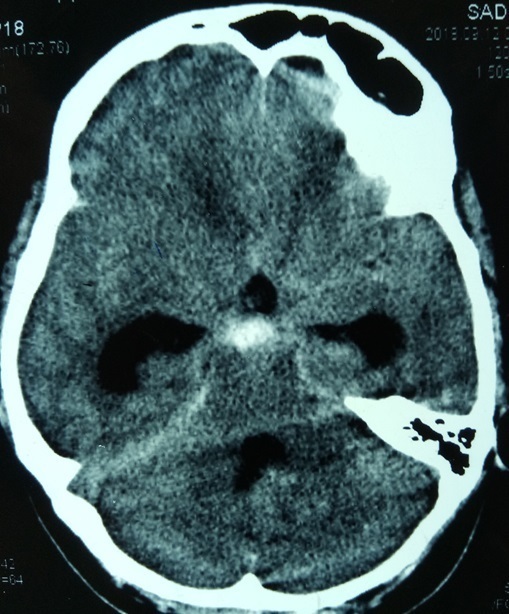
CT scan obtained at admission showing subarachnoid bleed in cisterns with hydrocephalus.

**Figure 2.**
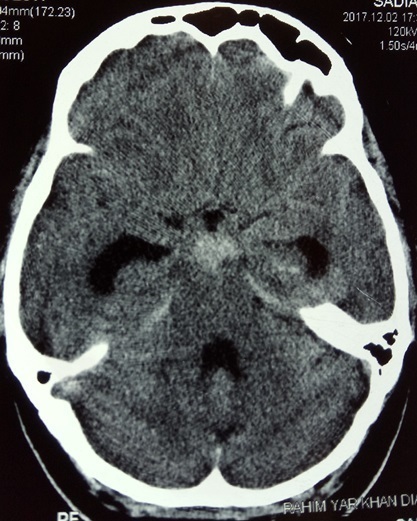
CT scan brain obtained 1 year back showing subarachnoid hemorrhage in interpeduncular cistern.

**Figure 3.**
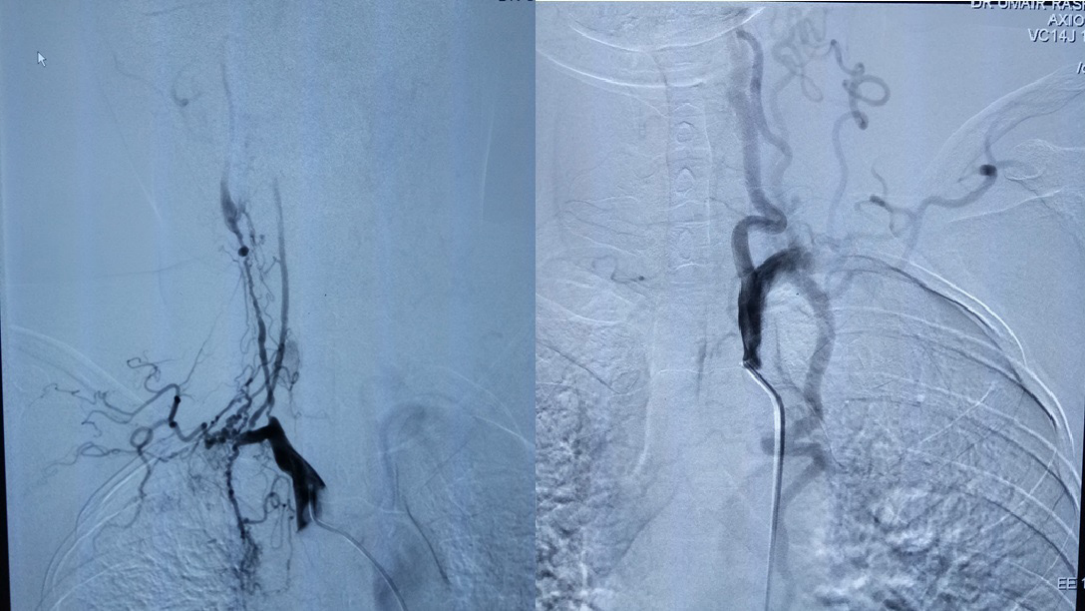
(a) Selective arch aortogram showing 90% stenosis of right ICA with distal dilatation prior tapering, right VA also appear to be stenotic from its origin. (b) Selective arch angiogram showing hypertrophied left VA, left subclavian occlusion from its proximal segment with collateral formation.

**Figure 4.**
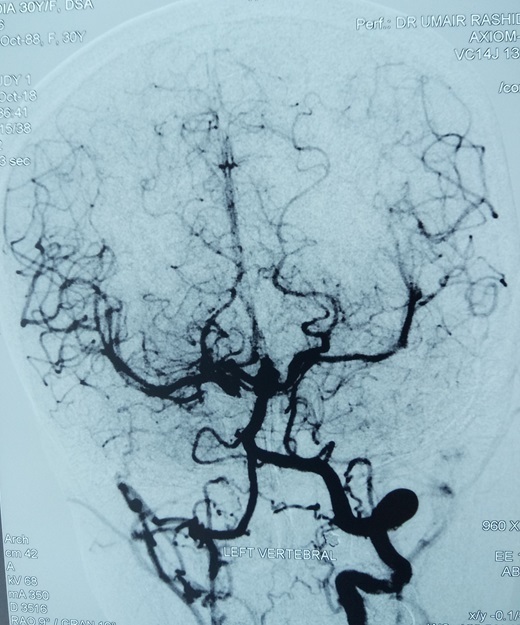
Anteroposterior and lateral view of selective left vertebral artery angiogram demonstrating basilar apex aneurysm of size (7.3 x 5.3mm). Vertebral arteries also giving collateral supply to middle cerebral artery and anterior cerebral artery territory.

**Figure 5.**
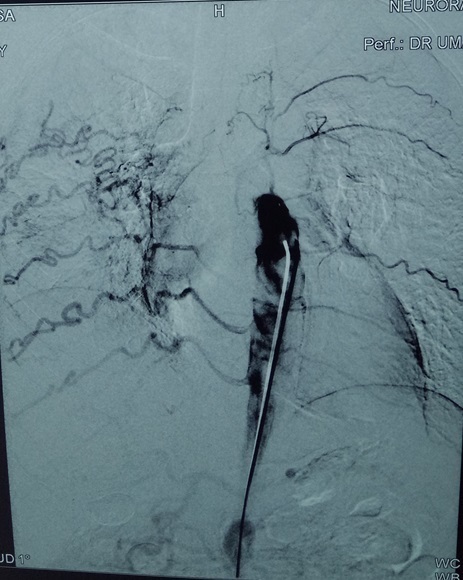
Selective aortogram demonstrating hypertrophied intercostal arteries bilaterally.

**Figure 6.**
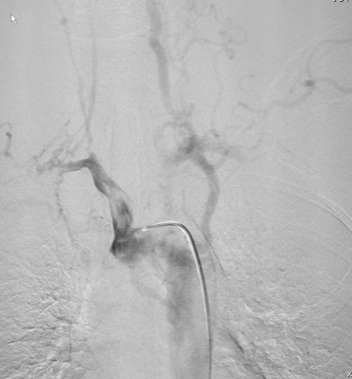
Selective arch aortogram showing bilateral subclavian stenosis at the proximal segment.

**Figure 7.**
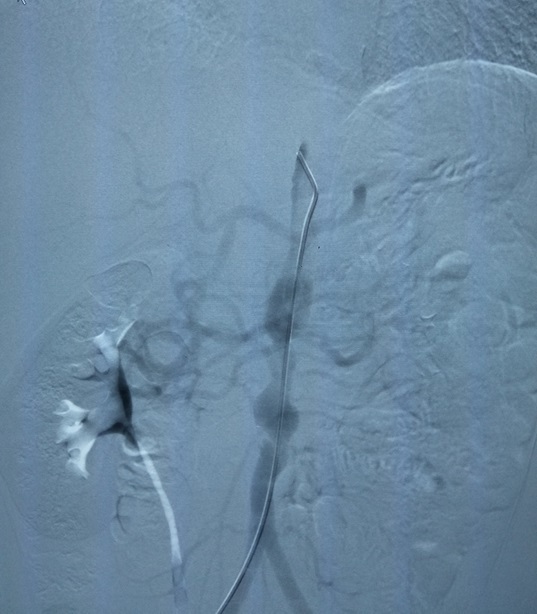
Distal aorta angiogram showing irregular distal aorta stenosis.

A CT brain was conducted that revealed subarachnoid hemorrhage with intraventricular extension (Figure 1). Similar findings were evident in previous CT scan brain which was done 1 year ago at the previous presentation of ictus (Figure 2). Laboratory investigation showed raised ESR upto 40 mm/h and C Reactive Protein was positive. Cerebral angiography and aortogram showed total occlusion of left common carotid artery from its origin and 90% occlusion of right common carotid artery from its origin with slightly dilated segment prior to its tapering (Figure 3). The sole arteries supplying the intracranial circulation were bilateral vertebral arteries and showed saccular basilar tip aneurysm of size 7.30 x 5.39 mm (Figure 4). Additional imaging showed bilateral occlusion of subclavian arteries from their initial segments. There were extensive collateral branches to intercostal arteries supplying both upper arms (Figures 5 and 6). The terminal aorta was irregular but bilateral renal arteries and other branches of aorta were normal (Figure 7). We started the patient on 20 mg prednisone resulting in a reduction of ESR as well as normalization of C Reactive Protein. Further the patient was referred for endovascular coil embolization as vertebral arteries were relatively straight and easy to access.

Coil embolization procedure was decided as line of approach for therapy. The navigation was straightforward from vertebral artery but unfortunately the patient had complication and third episode of subarachnoid hemorrhage night before the procedure and expired.

## Discussion

TA is a chronic, large vessel vasculitis of unknown etiology that typically effects aorta and its branches.^2^ TA is a rare disease and the etiology is unknown. It is thought that TA may run in families. Studies have shown increased incidence of TA in Asians who have a particular antigen on chromosome 6 (HLA-Bw52). Approximately 80–90% percent of cases presenting with TA are in females. The symptoms usually begin between 15 and 35 years although it can affect children as well.^4^

The general understanding is that an obscure stimulus triggers expression of heat shock protein-65 in the aortic tissue. This leads to acute inflammation, necrosis, new vessel formation, smooth muscle migration, intimal proliferation, and giant cell augmentation. Further, there is enlistment of B lymphocytes with creation of anti-endothelial, anti-cardiolipin, and anti-aortic auto-antibodies, which complements the inflammatory process.

Until 1988 no diagnostic or classification criteria has been developed for TA, when Ishikawa proposed a set of diagnostic criteria. In 1990, the American College of Rheumatology distributed the grouping criteria for seven vasculitidis including TA. Characterization criteria regarding children with TA was proposed by European League Against Rheumatism and the Pediatric Rheumatology International Trials Organization in 2005 and later approved in 2008. In order to build up a single classification system and an approved set of diagnostic criteria for systemic vasculitidis the Diagnostic and Classification Criteria in Vasculitis Study is a universal effort going on.

According to American College of Rheumatology Criteria the diagnosis depends upon fulfilment of at least three criterion; age less than 40 years, decreased brachial pulses, claudication of the extremities, a difference in the systolic pressure of upper limbs greater than 10 mmHg, murmurs in the subclavian and arch of aorta and angiographic evidence of changes in aorta and its branches.^5^

Cerebrovascular association have been reported in upwards of 24% cases of aorto-arteritis with occlusion, stenosis, aneurysms and arterial wall thickening seen for the most part in the extracranial carotid and vertebral circulation. The incidence of aneurysms in TA including the aorta and its significant branches is as high as 31.9%.^3^ The cerebral aneurysms and subarachnoid bleeding is an uncommon symptom with just 26 cases revealed in literature.^3^

Breno Bezerra et al reviewed literature and found 22 papers reporting 18 aneurysms in posterior circulation and 24 in anterior circulation in which the internal carotid artery, anterior communicating artery and basilar arteries were commonly effected, hence posterior circulation aneurysm are common in TA as was the case in this report.^3^

Other studies that failed to find evidence of active inflammation in cerebral aneurysm tissue, put forth a secondary theory that altered hemodynamics cause a secondary to the carotid artery and/or vertebral artery causing occlusion that is responsible for aneurysm formation in Takayasu arteritis.^6^

## Conclusion

In our case there was a delay in the diagnosis of the disorder and its treatment resulting in a poor outcome. Early diagnosis and hence this condition warrants prompt treatment and swift response.

TA is an extremely rare disease, with limited reported literature on the subject. Intracerebral TA presents as vessel occlusion, stenosis or aneurysm formation. In the case of subarachnoid hemorrhage as can be seen in this case report, aneurysm is the most likely outcome. Due to the inherent nature of the disease, major vessels are either stenosed or occluded which is why endovascular coiling is a difficult treatment modality. However, due to advancement in microcatheters and guide wires it has become possible when done with great care and major route planning pre-embolization.

## Key learning points

1. While intracranial vascular abnormalities in patients with Takayasu arteritis presenting with neurologic symptoms are not rare, with cerebral vasculitis seen in 7.8% of patients, and stroke secondary to large-vessel occlusion, in 3.9% of patients. However, intracranial aneurysms associated with Takayasu arteritis is rare with only 26 such reported cases.
2. Aneurysm formation and subarachnoid hemorrhages must be considered along with ischemic changes as neurological complications of Takayasu Arteritis otherwise this can go undiagnosed.
3. Previously due to stenosed and occluded vessels microsurgery was the only option but now since the advancement in the field of endovascular treatment we can now do safe and successful coil embolization of aneurysms.
